# Heterogeneity in the incidence of kidney replacement therapy across Europe: a benchmarking tool to improve clinical practice

**DOI:** 10.1093/ckj/sfae155

**Published:** 2024-05-21

**Authors:** Lucia Cordero, Alberto Ortiz

**Affiliations:** Department of Nephrology and Hypertension, IIS-Fundacion Jimenez Diaz UAM, Madrid, Spain; Department of Nephrology and Hypertension, IIS-Fundacion Jimenez Diaz UAM, Madrid, Spain; RICORS2040, Madrid, Spain; Departamento de Medicina, Facultad de Medicina, Universidad Autónoma de Madrid, Madrid, Spain

Despite the availability of kidney replacement therapy (KRT), chronic kidney disease (CKD) is among the fastest increasing global causes of premature death [1]. Longer life expectancy and population aging are contributing to an increase in the prevalence of CKD, the need for KRT and the median age at incident KRT, which increased from 67.1 to 68.4 years between 2012 and 2021 in territories reporting data to both iterations of the European Renal Association (ERA) Registry [1–3]. The heterogeneity in incident KRT across Europe was emphasized for the 2012 report [4] and remains topical in the 2021 ERA Registry Annual Report [3], which again showed large differences between territories. An in-depth analysis of these differences can be used as a benchmarking tool to identify best practices that improve kidney care.

In 2021, the incidence of KRT in participating territories was 145 per million people (pmp), with a majority of incident patients being ≥65 years of age (55%; median 68.2 years) [3]. However, the range of incidence and median age at incident KRT in individual territories was wide: 53–283 pmp (difference between highest and lowest incidence 230 pmp, 5.3-fold) and 56.0–74.5 years (difference 18.5 years, 1.33-fold). At the end of 2021, the prevalence of KRT was 1040 pmp and the median age of prevalent patients was 64 years [3]. Again, the range of prevalence and median age was wide at 304–2003 pmp (difference 1699 pmp, 6.6-fold) and 54.0–70.0 years (difference 16 years, 1.30-fold), respectively. This heterogeneity was also largely reflected in prior iterations of the ERA Registry [5, 6] and should be interpreted in the context of other differences in socio-economic and health indicators, such as gross domestic product (GDP) per capita, life expectancy at birth and others. The range for life expectancy at birth for participating territories was 69.6–83.9 years (difference 14.3 years, 1.20-fold); being even wider if life expectancy within regions of the same country is considered, the upper range increasing to 85.4 years when Spanish autonomous communities are considered individually [7, 8]. However, the heterogeneity at the age of incident KRT appears wide even when comparing it to other key health indicators and within a narrower range of GDP per capita.

In this regard, the use of registry data for benchmarking should be considered another benefit for healthcare authorities and the kidney health community of keeping and sharing records. When datapoints on median age at incident KRT and on KRT incidence from contributing territories are represented in a graph, the heterogeneity of clinical practice across Europe becomes evident while providing some early input into potential drivers of the differences (Fig. 1A) [3]. Additionally, representing the median values and interquartile range (IQR) for both variables identifies outliers in four quadrants that may be labelled high incidence/older age (Greece, Cyprus, Belgium, Catalonia, Israel), high incidence/younger age (Sfax region in Tunisia), low incidence/older age (Switzerland), low incidence/younger age (Ukraine, Montenegro, Lithuania, Latvia). Several factors may influence the outlier nature of data points and further research should identify and characterize these factors. Some are relatively unmodifiable, related to the general wealth of the country, potentially limiting access of the population to KRT because of budgetary reasons. This may result in a low incidence/younger age pattern. However, there are also actionable factors that may respond to increasing health education and awareness of the population and early identification and treatment of CKD. These actionable factors may contribute to high incidence/younger age patterns, even despite budgetary constraints: prevention and treatment of early disease tend to be cost-effective compared with treatment of kidney failure, especially if the main KRT modality is haemodialysis. Finally, more complex factors may contribute, as exemplified by societal attitudes that influence uptake of KRT in the elderly, such as attitudes of patients, physicians and healthcare authorities towards limitation of therapeutic efforts, choice of conservative care versus intervention and access to KRT. Unravelling them may provide insights into optimal approaches to initiating KRT, especially in the elderly and in those with comorbidities, in terms of quality of life and life expectancy, independent of budgetary considerations.

**Figure 1:**
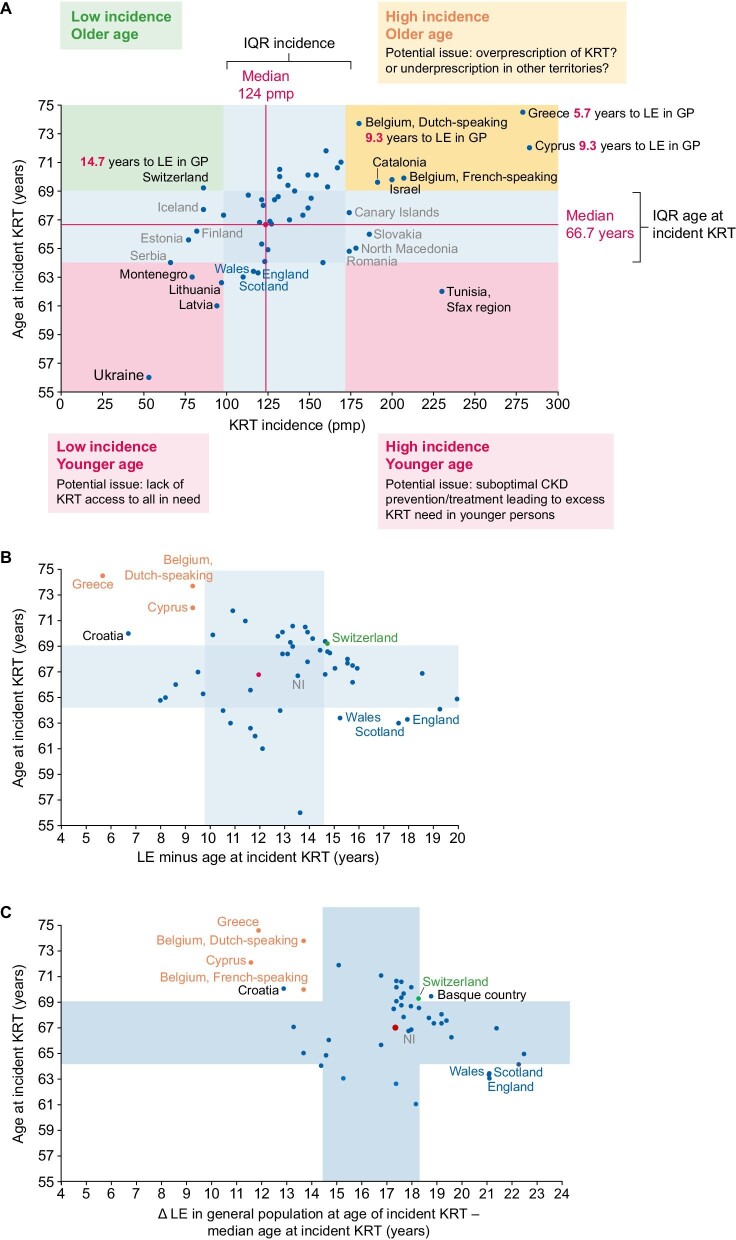
Geographical heterogeneity of incident KRT in the ERA Registry Annual Report 2021. **(A)** Unadjusted incidence of KRT in 2021 on day 1 versus median age at initiation of KRT. Median values for both variables for participating countries are represented as a red datapoint. Median and IQR were calculated from data for the countries presented in Table 1 of reference 1, except for Spain. Although Spanish autonomous communities are presented as data points in the graph, only the Spain (all) data were used to calculate the median and IQR for each variable. Q1 and Q3 were used as the interquartile area for each variable (in blue). Territories with values outside the combined IQR for both variables were considered outliers for the purpose of this figure and country names are presented in black. Additional labels in grey identify the names of countries outside the IQR for incidence, while labels in blue indicate countries below the IQR for age at incident KRT. Potential interpretation of the four outlier quadrants is provided as food for thought and debate. **(B)** Additionally, the gap between life expectancy at birth in the general population (2021 data from Eurostat statistics and World Bank Data) [7, 8] and age at incident KRT was calculated for each country as was the median and IQR of this new variable. In panel A, the value for the numerical gap between life expectancy (LE) at birth in the general population (GP) and age at incident KRT is shown in red in labels for the countries that are outliers at both panel A and panel B (defined as outside the IQR of 9.8–14.6 years). Panel B (in orange and green) the names of outlier countries from panel A that are also outliers in panel B. Names of countries that are only outliers in panel B are identified in black or blue, depending on their relationship to the IQR. Since three of these panel-B-only outliers are UK countries, the fourth UK country, Northern Ireland (NI, not an outlier) is shown in grey for comparison. **(C)** Same procedure and colour coding as in panel B, but using as reference life expectancy in the general population at the median age of incident KRT in each country (2021 data from Eurostat statistics). Note that life expectancy in the general population is now longer (panels B and C are drawn to the same scale; Ukraine missing from panel C). Three UK countries remain in one extreme and now cluster closer, while on the other extreme, both Belgium regions now cluster closer. Data in panels A and B are presented for Albania, Austria, Belgium (Dutch-speaking and French-speaking), Bosnia and Herzegovina, Croatia, Cyprus, Denmark, Estonia, Finland, France, Greece, Iceland, Israel, Italy (7 of 20 regions, all as a single data point), Kosovo, Latvia, Lithuania, Montenegro, North Macedonia, Norway, Romania, Serbia, Slovakia, Spain (all and individual datapoints for each autonomous community), Sweden, Switzerland, The Netherlands, Tunisia (Sfax region), Ukraine, England, Northern Ireland, Scotland and Wales. In panel C, some countries are missing because of missing life expectancy data. However, the IQR for age at incident KRT is maintained as in panels A and B to better allow comparisons.

An initial approach to unravelling the drivers of heterogeneity may focus on assessing territories with outlier values and relating these outlier values to variables that may influence them, such as life expectancy in the general population. Among participating territories, life expectancy at birth in the general population is also very variable [7, 8]. Estimation of the median and IQR for the gap between life expectancy at birth in the general population and age at incident KRT will allow identification of outliers for this new metric that considers that the same chronological age may have different meanings regarding health for different territories (Fig. 1B). Among outlier territories for the combination of incidence and mean age at incident KRT, Greece, Cyprus and Dutch-speaking Belgium on the one hand and Switzerland on the other are also outliers falling on different sides of the spectrum of the gap between life expectancy in the general population and age at incident KRT. The first three countries initiate KRT at median ages closer to the life expectancy in the general population than most other countries, the gap being 5.7 years for Greece and <10 years for Cyprus and Dutch-speaking Belgium. These countries are also outliers in the sense that KRT is initiated more frequently in elderly individuals, applying a local concept of elderly (i.e. being closer to the local life expectancy at birth in the general population). In other words, they appear to have a more open attitude towards offering (physicians and payers) and accepting (patients) KRT in the elderly (with the ‘elderly’ concept adapted to local standards) than other European countries. Whether this is more beneficial to patients and the healthcare system than standard practice is a key research question. In contrast, Switzerland initiates KRT at a median age 14.7 years younger than the country’s life expectancy at birth (i.e. the practice in Switzerland would be similar to other European countries such as Spain and The Netherlands in terms of age at initiation of KRT adjusted for life expectancy in the country), and the lower incidence of KRT in Switzerland may partially reflect the practice of less frequent initiation of KRT in the elderly (defining elderly as close to the local life expectancy at birth) than in Greece, Cyprus and Dutch-speaking Belgium, independent of the potential influence of other factors such as differences in the prevalence or speed of progression of CKD. In contrast, countries such as Ukraine, which has the lowest age at the start of KRT, is on par with the rest of Europe regarding the gap between age at incident KRT and local life expectancy. They start KRT at a younger age than in other countries (potentially implying a lack of access of the elderly to KRT), but life expectancy at birth is also lower (i.e. they start KRT in the elderly, defining elderly as close to the local life expectancy at birth). Three UK countries also have a relatively young median age at incident KRT (below the IQR for this metric; Fig. 1A), associated with a very large gap in local life expectancy at birth (Fig. 1B), suggesting that the elderly (‘elderly’ concept adapted to local standards) are not offered or are rejecting KRT to a greater extent than in other countries, including Northern Ireland. In these UK countries, people appear to be considered ‘elderly’ at a younger age (relative to local life expectancy) than in other ERA Registry countries.

When using life expectancy of the general population at the median age of KRT initiation as a reference in each country instead of life expectancy at birth, the gap between age at incident KRT and life expectancy is greater and countries tend to cluster closer together. Specifically, UK countries and Belgium cluster closer together, suggesting that this may represent a better metric (Fig. 1C).

Statements on the heterogeneity of age at incident KRT and KRT incidence in the present text do not imply a judgment regarding what should be considered best practice. Only detailed analyses of quality of life on and off KRT and survival on and off KRT for elderly persons with kidney failure will provide answers, and best practices may differ by country according to local population characteristics, quality of conservative care and KRT, opportunity for transplantation and other factors. In this regard, a single-year dataset is not enough to draw firm conclusions but illustrates potential analyses to be performed in larger multiyear datasets resulting from averaging age at incident KRT and incidence of KRT over longer time periods (e.g. 10 years). In this regard, reliability of the data is key, and their interpretation would be heavily jeopardized if the collected data were unreliable. Any analysis of incident KRT should also address heterogeneity in pre-emptive kidney transplantation (range 0–17.3% of incident KRT in 2021) [3, 9].

In conclusion, the ERA Registry Annual Report 2021 continues to provide a wealth of information that may contribute to optimize the indication of KRT, especially among the elderly, in order to maximize benefit for patients while contributing to sustainable healthcare. In this regard, benchmarking should be one of the key aims of ERA Registry analyses and a holistic benchmarking strategy that integrates key demographic and health variables from participating countries should be designed and implemented [10].

## References

[1] Ortiz A, Asociación Información Enfermedades Renales Genéticas (AIRG-E), European Kidney Patients' Federation (EKPF), Federación Nacional de Asociaciones para la Lucha Contra las Enfermedades del Riñón (ALCER), Fundación Renal Íñigo Álvarez de Toledo (FRIAT), Red de Investigación Renal (REDINREN), Resultados en Salud 2040 (RICORS2040), Sociedad Española de Nefrología (SENEFRO) Council, Sociedad Española de Trasplante (SET) Council, Organización Nacional de Trasplantes (ONT). RICORS2040: the need for collaborative research in chronic kidney disease. Clin Kidney J 2021;15:372–87. 10.1093/ckj/sfab170 35211298 PMC8862113

[2] Pippias M, Stel VS, Abad Diez JM et al. Renal replacement therapy in Europe: a summary of the 2012 ERA-EDTA Registry Annual Report. Clin Kidney J 2015;8:248–61. 10.1093/ckj/sfv014 26034584 PMC4440462

[3] Boerstra BA, Boenink R, Astley ME et al. The ERA Registry Annual Report 2021: a summary. Clin Kidney J 2024;17:sfad281. 10.1093/ckj/sfad281 38638342 PMC11024806

[4] Gonzalez-Espinoza L, Ortiz A. 2012 ERA-EDTA Registry Annual Report: cautious optimism on outcomes, concern about persistent inequalities and data black-outs. Clin Kidney J 2015;8:243–7. 10.1093/ckj/sfv035 26034583 PMC4440478

[5] Astley ME, Boenink R, Abd ElHafeez S et al. The ERA Registry Annual Report 2020: a summary. Clin Kidney J 2023;16:1330–54. 10.1093/ckj/sfad087 37529647 PMC10387405

[6] Boenink R, Astley ME, Huijben JA et al. The ERA Registry Annual Report 2019: summary and age comparisons. Clin Kidney J 2021;15:452–72. 10.1093/ckj/sfab273 35211303 PMC8862051

[7] Eurostat . Statistics explained. Life expectancy at birth, 1980–2021. https://ec.europa.eu/eurostat/statistics-explained/index.php?title=File:Table01_Life_expectancy_at_birth_2021.png [accessed 19 December 2023].

[8] World Bank . Life expectancy at birth, total (years). https://data.worldbank.org/indicator/SP.DYN.LE00.IN [accessed 19 December 2023].

[9] Kramer A, Boenink R, Mercado Vergara CG et al. Time trends in preemptive kidney transplantation in Europe: an ERA Registry study. Nephrol Dial Transplant 2024:gfae105. 10.1093/ndt/gfae105 38724446 PMC11648960

[10] Ortiz A . Benchmarking CKD: incidence of CKD in a European country with low prevalence of CKD and kidney replacement therapy. Clin Kidney J 2022;15:1221–5. 10.1093/ckj/sfac074 35756737 PMC9217648

